# Effect of Kinesiology Taping on Breast Cancer-Related Lymphedema: A Randomized Single-Blind Controlled Pilot Study

**DOI:** 10.1155/2013/767106

**Published:** 2013-11-27

**Authors:** A. Smykla, K. Walewicz, R. Trybulski, T. Halski, M. Kucharzewski, C. Kucio, W. Mikusek, K. Klakla, J. Taradaj

**Affiliations:** ^1^Department of Physiotherapy Basics, Academy of Physical Education in Katowice, Mikolowska 72 Street, 40-065 Katowice, Poland; ^2^Department of Medical Biophysics, Medical University of Silesia in Katowice, Medykow 18 Street, 40-752 Katowice, Poland; ^3^Department of Physiotherapy, Provita Clinic in Zory, Boczna 6 Street, 44-240 Zory, Poland; ^4^Department of Physiotherapy, Public Higher Professional Medical School in Opole, Katowicka 68 Street, 40-060 Opole, Poland; ^5^Department of Descriptive and Topographic Anatomy, Medical University of Silesia in Zabrze, Jordana 19 Street, 41-808 Zabrze, Poland; ^6^Department of Physiotherapy in Internal Medicine, Academy of Physical Education in Katowice, Mikolowska 72 Street, 40-065 Katowice, Poland; ^7^Department of General Surgery and Gastroenterology, Medical University of Silesia in Bytom, Zeromskiego 7 Street, 41-902 Bytom, Poland

## Abstract

The aim of the study was to assess the efficacy of Kinesiology Taping (KT) for treating breast cancer-related lymphedema. Sixty-five women with unilateral stage II and III lymphedema were randomly grouped into the KT group (K-tapes, *n* = 20), the Quasi KT group (quasi K-tapes, *n* = 22), or the MCT group (multilayered compression therapy group, *n* = 23). Skin care, 45 min pneumatic compression therapy, 1 h manual lymphatic drainage, and application of K-tape/Quasi K-tapes/multilayered short-stretch bandages were given every treatment session, 3 times per week for 1 month. Patient evaluation items included limb size and percentage edema. Comparing the changes in K-tapes with quasi K-tapes changes, there were no significant differences (*P* > 0.05). The edema reduction of multilayered bandages was much better than in results observed in taping groups. The KT appeared to be ineffective at secondary lymphedema after breast cancer treatment. The single-blind, controlled pilot study results suggest that K-tape could not replace the bandage, and at this moment it must not be an alternative choice for the breast cancer-related lymphedema patient. The trial is registered with ACTRN12613001173785.

## 1. Introduction

Lymphedema is a chronic and progressive condition resulting from an abnormality or damage to the lymphatic system. It is marked by an abnormal increase of tissue proteins, edema, chronic inflammation, and fibrosis. Secondary lymphedema is caused by multiple factors related with lymphatic stasis, such as tumor lymph node infiltration, lymph node dissection, radiotherapy, trauma, and infection. Upper limb lymphedema occurs in 24–49% of the cases with total mastectomy and in 2.4–49% of the cases with axillary lymph node dissection [1, 2].

In Western Europe [3], upper limb secondary lymphedema has been reported in 22% of patients after breast cancer therapy. Lymphedema occurs when there is an imbalance due to reduced lymph transport capacity which leads to interstitial fluid and protein accumulation. It further leads to chronic inflammation and fibrosis caused by the secondary proliferation of neutrophils, macrophages, and fibroblasts and accumulation of collagen.

Physical therapy is a common management for lymphedema. A program combining skin care, manual lymphatic drainage, exercise, and compression therapy (multilayered bandage, intermittent pneumatic compression) is recognized as the best practice in lymphedema management. There have been numerous prospective investigations with different treatment frequency and duration showing the effect of physical therapy, which has been accepted as a standard “gold” therapy for many years [4–8].

However, standard care and management can have significant economic consequences. Bandage changes and expensive compression hosiery drain the available resources. Well-documented, promising, and inexpensive methods from alternative medicine are still needed [9–13].

Kinesiology Taping (KT) for lymphatic drainage is a new choice in the field of physical and alternative therapy. The material used for the Kinesio tape and the original concept of the taping technique were introduced by Dr. Kenso Kase in 1973. K-tape had been designed to allow 30–40% longitudinal stretch. It is composed of 100% cotton fibers and acrylic heat sensitive glue. Development of the technique for its administration is still ongoing. Dr. Kase claimed that applying K-tape would have physiological effects including decreasing pain or abnormal sensation, supporting the movement of muscles, removing congestion of lymphatic fluid or hemorrhages under the skin, and correcting misalignment of joints. After applying K-tape, the taped area will form convolutions to increase the space between the skin and muscles. Once the skin is lifted, the flow of blood and lymphatic fluid is promoted. Other advantages are that a patient can take a shower without taking the tape off since it is waterproof. Patients can wear it from 1 to 4 days and even longer if it is applied on the back or buttock area [14, 15].

Many practitioners use it in clinical practice in European countries, and it has a beneficial effect. However, there is insufficient evidence for its clinical effects on lymphedematous limbs. The aim of the study was to assess the efficacy of Kinesiology Taping (KT) for treating breast cancer-related lymphedema. The endpoints were the reduction of limb volume and percentage edema size after a month's therapy.

## 2. Materials and Methods

The Research Ethics Committee from the Academy of Physical Education in Katowice, Poland, approved this study (national registration number 1605/12/2012). The trial is registered in the Australian New Zealand Clinical Trials Registry with ID number ACTRN12613001173785.

### 2.1. Settings and Participants

**Figure 1:**
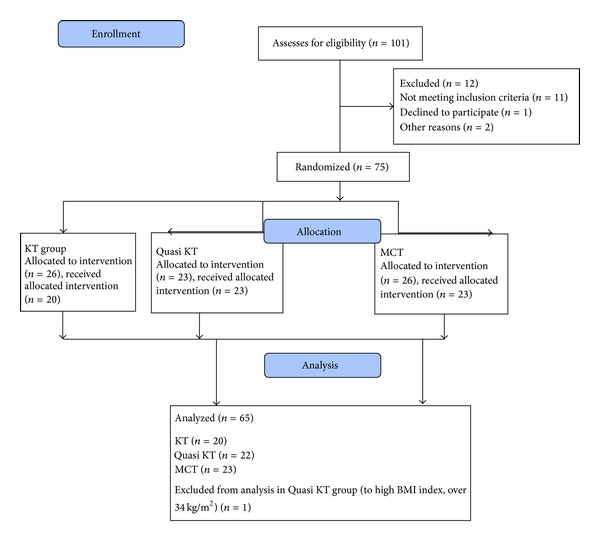
Flow diagram of the study.

The study was performed at the Provita Clinic in Zory and Limf-Med Hospital in Chorzow, Poland, from December 2012 to August 2013. Participating women met the following inclusion criteria: (1) unilateral breast cancer-related lymphedema for at least one year, (2) moderate-to-severe lymphedema (stages II and III of upper limb edema, the volume difference between affected and healthy extremity with being more than 20%), (3) lack of chemo- or radiation therapy for at least 6 months, and (4) good compliance and willingness to sign the written consent form. Subjects with the following conditions were not allowed to participate or were excluded from the study: (1) active cancer or disease that might lead to swelling and presently taking diuretic therapy or other lymphedema-influencing drugs, (2) skin disease, (3) irremovable bracelet or ring, (4) marked restriction of active range of motion in the affected upper extremity, (5) the presence of a pacemaker, heart disease, pregnancy, metallic devices in the limb to be treated, infectious disease, epilepsy, cartilage growth, thrombophlebitis, arterial hypertension, or metastases, which are the treatment contraindications, and (6) the presence of mental, sensorial, or language problems, which could make cooperation difficult (more details in Figure 1).

### 2.2. Randomization and Intervention

Participants were randomly allocated to the groups. Computer-generated random numbers were sealed in sequentially numbered envelopes, and the group allocation was independent of the time and person delivering the treatment. The physician (main coordinator) who allocated the patients to groups had 75 envelopes, each containing a piece of paper marked with either group KT, Quasi KT, or MCT. The physician would select and open an envelope in the presence of a physiotherapist to see the symbol and would then direct the patient to the corresponding group. A clinical nurse collected the data and coded them into an Excel database. The “blinded” results were transferred to a STATISTICA version 10.0 (StatSoft Inc., Poland) database by a technician. The research coordinators had no contact with and could not identify the patients.

**Figure 2:**
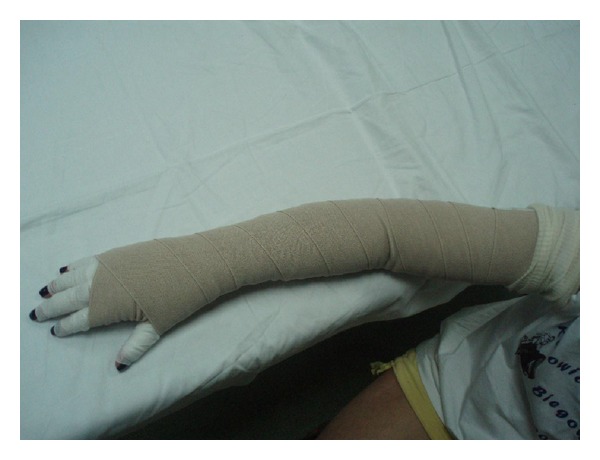
Multilayered compression bandaging.

**Figure 3:**
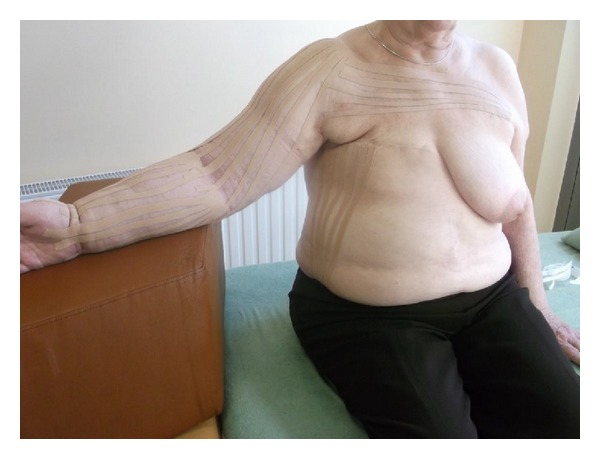
Kinesiology Taping application.

**Figure 4:**
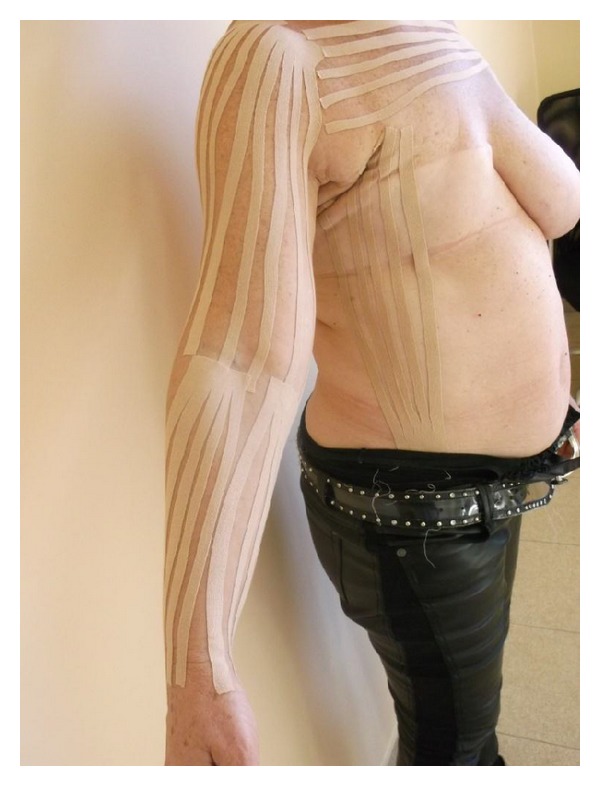
K-tapes technique.

Subjects from all groups received a routine treatment, including skin care, 45 min pneumatic compression therapy in use of the DL1200 device (at pressure 90 mmHg, 12 chambers arm overlapping cuff, hold time 3 seconds with no interval), 1 h manual lymphatic drainage, and application of multilayered short-stretch bandages (50–60 mmHg). The tape groups (KT and Quasi KT groups also received standard therapy, but K-tapes were used instead of bandages). Each of the groups was treated 3 times weekly (bandages or K-tapes were applied and changed on Mondays, Wednesdays, and Fridays) for in the 4-week intervention period. One physical therapist (PT) provided treatment. The program was standardized, following the same protocol for lymphatic drainage to the anterior trunk, posterior trunk, and affected arm, always mowing fluid from the affected side toward the unimpaired side, after lymphatic drainage and before either the short-stretch bandages (Figure 2) or the Kinesiology Taping application (Figure 3). Both bandages and K-tapes (Figure 4) were applied by the by the physical therapist.

In KT group, the fan tape anchor started at the anterior aspect of the hand with no tension. The tails of the tape were applied to the anterior, medial, and posterior aspects of the forearm and arm with 5–15% tension and then on anterior part of chest. The tapes were left on the patient's skin for the next three days. In Quasi KT group, we used tapes without therapeutic effects-common surgical plasters stuck using the same methodology as in KT group.

In MCT group, we used 4-layered compression bandaging. The first layer was applied to the skin directly with Tubula orthopedic sleeve. Then a supporting bandage Matoplast was applied to the fingers and on hand. Another layer was cotton Rolta-Soft covering the whole limb. The external layer consisted of short-stretch Hartmann bandages.

The following research was a single-blind, controlled, randomized clinical study. The experiment design, methodology, and treatment parameters were programmed by coordinators (physiotherapist, general surgeon, oncologist, and an internist). Standard care, optoelectronic measurements, and data collection were provided by a nurse. The KT/Quasi KT was performed by a physiotherapist. The final statistical analysis was performed by a technician.

### 2.3. Outcomes Assessment

**Figure 5:**
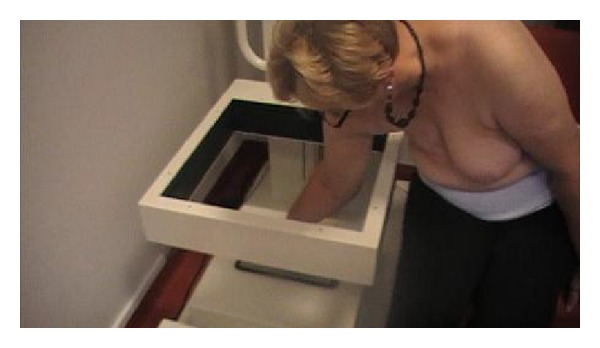
Optoelectronic limb volume measurement.

**Figure 6:**
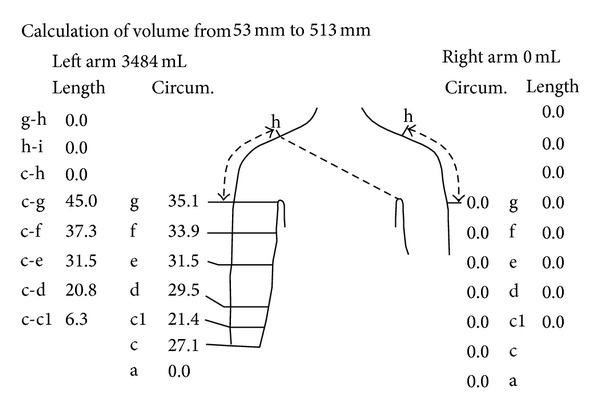
Graphical presentation of optoelectronic measurement.

To assess the volume of limb, we used an optoelectronic Perometer 40 T, cooperating with a personal computer. This method allowed us to estimate the volume of the measuring error for only 0.5%. The assessment technique was based on a special ring, equipped with a system of 378 LED diodes (emitting the infrared radiation). Within the ring were also the optical sensors that receive electromagnetic stimuli. In the course of measuring the limb was located inside the ring on the diode-sensor lines. The registered light pulses on the detectors were turned into electronic signals. The ring was moved during measurement to cover the entire limb (Figure 5). Measurements of the limb volume (both affected and healthy upper limb) were made for all three groups of patients before and after therapy (Figure 6).

### 2.4. Statistical Analysis

To compare the individual parameters that characterized the study groups, the nonparametric Kruskal-Wallis test for countable variables and the chi-squared test (*χ* ^2^) for categorical variables were used. The nonparametric matched pair Wilcoxon test was used to compare the within-group results before and after therapy. The Kruskal-Wallis analysis of variance (*post hoc* Tukey's test) was used to evaluate differences in the changes between the groups in the limb volume and edema values. Two-sided results (*P* < 0.05) were considered to be statistically significant.

## 3. Results

In total, 75 individuals were qualified to participate in the treatment. Six patients dropped out from the study during therapy in the KT group (one patient chose to discontinue treatment and withdrew from the study for personal reasons—taking care in the home of her daughter suffering from scarlet fever—four women had skin allergy after K-tapes, and one woman had a heart attack). Three patients from MCT group had complications unrelated to the treatment and were directed to other hospitals (one patient died of brain stroke) before the final observation. One patient in the Quasi KT (placebo group) was excluded from the analysis (BMI over 34 kg/m^2^, which was too high and significantly increased the SD; this increase could have seriously affected the reliability of both the nonparametric Kruskal-Wallis analysis of variance and the final conclusions).

Of the 65 patients who completed the study protocol (and were analyzed) and had stage II and stage III of secondary lymphedema of upper limb (Table 1), the average volume of the affected extremities in women from group KT was 9414.01 cm^3^ and decreased after treatment to 8051.15 cm^3^ (*P* = 0.002). The average volume of affected limb in women from Quasi KT group was 9621.33 cm^3^ and decreased after treatment to 8041.02 cm^3^ (*P* = 0.002).

In turn, the average volume of the affected limbs in women from MCT group was 10089.41 cm^3^ and after treatment it was 5021.22 cm^3^ (*P* = 0.000001).

**Figure 7:**
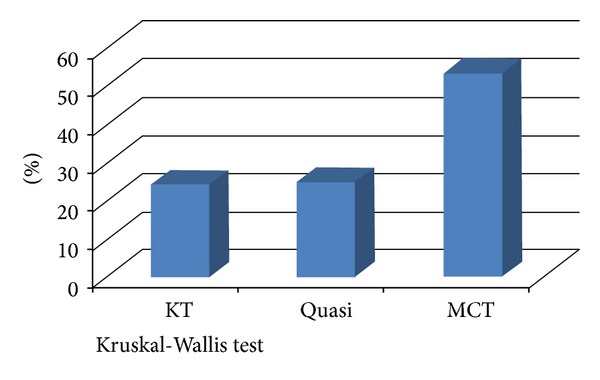
Comparing percentage edema reduction between groups. KT versus MCT group (24.45% versus 53.21%, *P* = 0.02). Quasi KT versus MCT group (24.78% versus 53.21%, *P* = 0.02). KT versus Quasi KT group (24.45% versus 24.78%, *P* = 0.455).

In the following study, we observed that the most significant decrease of edema was in patients undergoing multilayered compression bandaging. Results in patients undergoing K-tapes were similar to those obtained in the single-blind placebo (Quasi KT) group (Table 2 and Figure 7).

## 4. Discussion

The KT has been suggested as a promising treatment option for acute sport injuries [16], musculoskeletal disorders [17, 18], and also edema like venous and lymphedema [19–21], but there are still many controversies connected with methodology, application technique, and pressure values.

For example, Olszewski [22] maintains that lymphatics contract rhythmically with a frequency depending on the volume of inflowing tissue fluid. In regions with high capillary filtration rate and tissue fluid formation the frequency is high. The recorded pressures at rest, irrespective of whether in the lying or upright position, with free proximal flow (lateral pressure) range between 7 and 30 mmHg and during finger flexing between 10 and 30 mmHg. The pulse amplitude is from 3 to 20 mmHg and 5–17 mmHg, respectively. The pulse frequency is from 0.6 to 6/min and from 2 to 8/min, respectively. The resting end pressures with obstructed flow (e.g., corresponding to lymphatic obstruction in postsurgical lymphedema) range between 15 and 55 mmHg and during foot flexing from 15 to 50 mmHg. The pulse amplitude is from 3 to 35 mmHg and from 3 to 14 mmHg, respectively. The pulse frequency is from 2.5 to 10/min and from 3 to 12/min, respectively.

It means that external low pressure value during compression procedures (under 50 mmHg) has no effect on lymph pressures. In obstructive lymphedema only few lymphatic collectors remain patent. The recorded pressures during rest range from 5 to 45 mmHg depending on the remaining contractility force of the damaged lymphatic musculature. During calf muscular contractions pressures are generally low ranging from 10 to 25 mmHg, although well-conducted compression may in some cases generate pressures of above 60 mmHg. The author [22] recommends only high pressure range: 50–60 mmHg. In his opinion, lower values are useless, which is similar to our view of the results arising from the study, because K-tapes cannot induce higher external pressure than 15–20 mmHg.

We could find only one reliable meta-analysis presented by researchers from Israel [20], whose systematic review article tried to assessed the effects of therapeutic Kinesiology Taping on pain and disability in participants suffering from musculoskeletal, neurological, and lymphatic pathologies. Four online databases (CINAHL, Cochrane Library, MEDLINE, and PEDro) were comprehensively searched from their inception through March 2012. The initial literature search found 91 controlled trials. Following elimination procedures, 26 studies were fully screened. Subsequently, 12 met our inclusion criteria. The final 12 articles were subdivided according to the basic pathological disorders of the participants' musculoskeletal (*n* = 9), neurological (*n* = 1), and lymphatic (*n* = 2) systems. As to the effect on musculoskeletal disorders, moderate evidence was found supporting an immediate reduction in pain while wearing the KT. In 3 out of 6 studies, reduction of pain was superior to that of the comparison group. However, there is no support indicating any long-term effect. Additionally, no evidence was found connecting the KT application to elevated muscle strength or long-term improved range of movement. No evidence was found to support the effectiveness of KT for neurological conditions. As to lymphatic disorders, inconclusive evidence was reported. Although KT has been shown to be effective in aiding short-term pain, there is no firm evidence-based conclusion of the effectiveness of this application on the majority of movement disorders within a wide range of pathologic disabilities. In the authors' opinion, more research is clearly needed.

However (in only one found clinical trial in Pubmed and MEDLINE), Tsai et al. [21] presented a study about the positive effects of KT. The purpose of this experiment was to compare the treatment and retention effects between standard physical therapy combined with pneumatic compression and modified physical activity, in which the use of a short-stretch bandage was replaced by the use of Kinesiology Taping combined with pneumatic compression. The study results suggest that K-tapes could replace the bandage in therapy and could be a good alternative for patients with poor short-stretch bandage compliance. In our opinion, the mean weakness of this cited study are as follows: the Korean authors applied only single-layered compression therapy (15–20 mmHg, which is not enough to treat any kind of lymphedema) and there was a lack of estimation of the placebo effect in this article.

In the literature, there is a lack of well-conducted, randomized, controlled studies with KT and breast cancer-related lymphedema. It means that we will have to conduct our study. To this moment, we analyzed only a pilot group of women with secondary lymphedema after breast cancer treatment; further studies will be provided.

## 5. Conclusion

The KT appeared to be ineffective at secondary lymphedema after breast cancer treatment. The single blind, controlled pilot study results suggest that K-tape could not replace the bandage, and at this moment it must not be an alternative choice for the breast cancer-related lymphedema patient.

## Figures and Tables

**Table 1:** Characteristics of patients.

	Group KT	Group Quasi KT	Group MCT	*P*
Number of women**	20	22	23	0.784
Age (years)**				
Range	44–80	39–81	42–81	0.835
Average	67.34	65.43	66.45	
Median	66.11	63.89	67.81	
SD	12.03	13.13	11.99	
Total mastectomy*	20	22	23	0.784
Number of patients with adipositas* (BMI > 30 kg/m^2^)	7	7	6	0.812
Smokers*	7	8	7	0.812
Chemotherapy*	12	10	11	0.812
Radiation therapy*	15	13	13	0.788
Side of lymphedema*				
Right	8	10	10	0.679
Left	12	12	13	
Duration of lymphedema (months)**				
Range	12.2–63.6	12.3–46.6	15.3–33.8	0.621
Average	22.12	22.78	20.03	
Median	22.02	22.52	21.67	
SD	12.56	13.01	13.02	
Lymphedema severity** (% compared to healthy limb)				
II stage (20–40%)	15	16	16	0.788
III stage (40–60%)	5	6	7	

*χ ^2^ test.

**Kruskal-Wallis test.

**Table 2:** Results in percentage edema (affected upper limb compared to healthy limb volume and expressed in percent).

	Group	Average ± SD		*P*
		Before therapy	After therapy	
Decrease of edema (%)	KT	31.03 ± 28.17	25.03 ± 23.08	**0.005**
	Quasi KT	30.28 ± 30.12	24.47 ± 23.55	**0.005**
	MCT	31.07 ± 29.30	14.02 ± 10.03	**0.000003**

Wilcoxon test.
